# Supplementary material for: Using CombiCells, a platform for titration and combinatorial display of cell surface ligands, to study T-cell antigen sensitivity modulation by accessory receptors
Source: EMBO J. 2023 Dec 18;43(1):7. doi: 10.1038/s44318-023-00012-1 (PMC10897201; doi:10.1038/s44318-023-00012-1)
Supplement: Supplementary file 1 — Appendix [file 44318_2023_12_MOESM1_ESM.pdf]

Appendix

Appendix Figure S1 p. 1
Appendix Figure S2 p. 2
Appendix Figure S3 p. 3
Appendix Figure S4 p. 4
Appendix Figure S5 p. 5

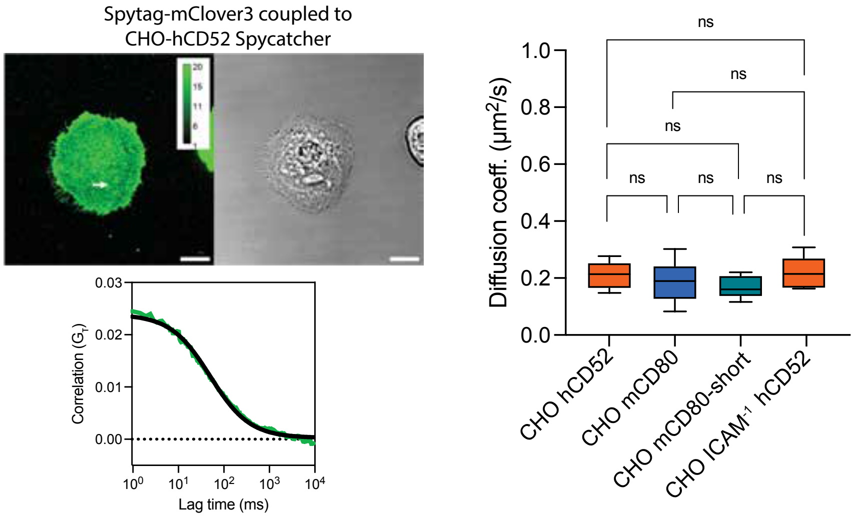

Appendix Figure S1: **The diffusion coefficient of surface Spycatcher.**

Scanning fluorescent correlation spectroscopy (sFCS) is used to determine the diffusion coefficient of surface Spycatcher. Representative confocal image of Spytag-mClover3 coupled to surface Spycatcher acquired in photon-counting mode (top left). Calibration bar indicates photons per pixel and white arrow indicates position of the sFCS line (scale bar = 10μm). Representative spatially-averaged auto-correlation from a single cell is fit to determine the diffusion coefficient (bottom left; data - green, model fit - black). The diffusion coefficients of Spytag-mClover3 coupled to the indicated surface Spycatcher on the indicated CHO-K1 cells (right).

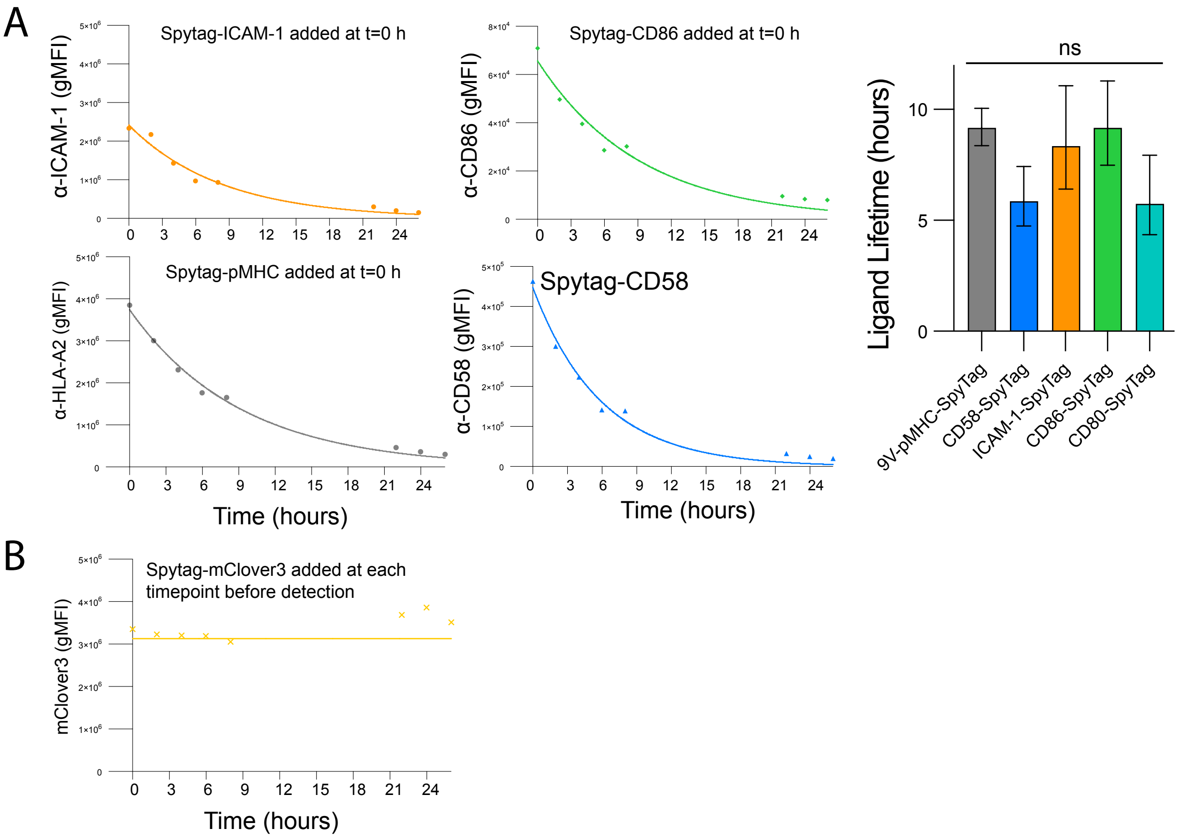

Appendix Figure S2: **Lifetime of Spytag-ligands coupled to surface Spycatcher on CombiCells detected using ligand-specific antibodies.**

(A) The indicated ligand was coupled ([ligand] = 0.5 μM) and detected using a conformational specific antibody in flow cytometry (left). An exponential fit produced an estimate for the ligand lifetime (right).

(B) The total surface level of Spycatcher was detected at each time point by coupling Spytag-mClover3 at each time point immediately before flow cytometry.

Data information: The mean of N=4 independent experiments is shown in (A,B) with the fitted lifetime and estimated error from the fit shown in (A, right panel). A F-test is used to determine a p-value for the null hypothesis that a single lifetime can fit all the data (ns = p-value>0.05).

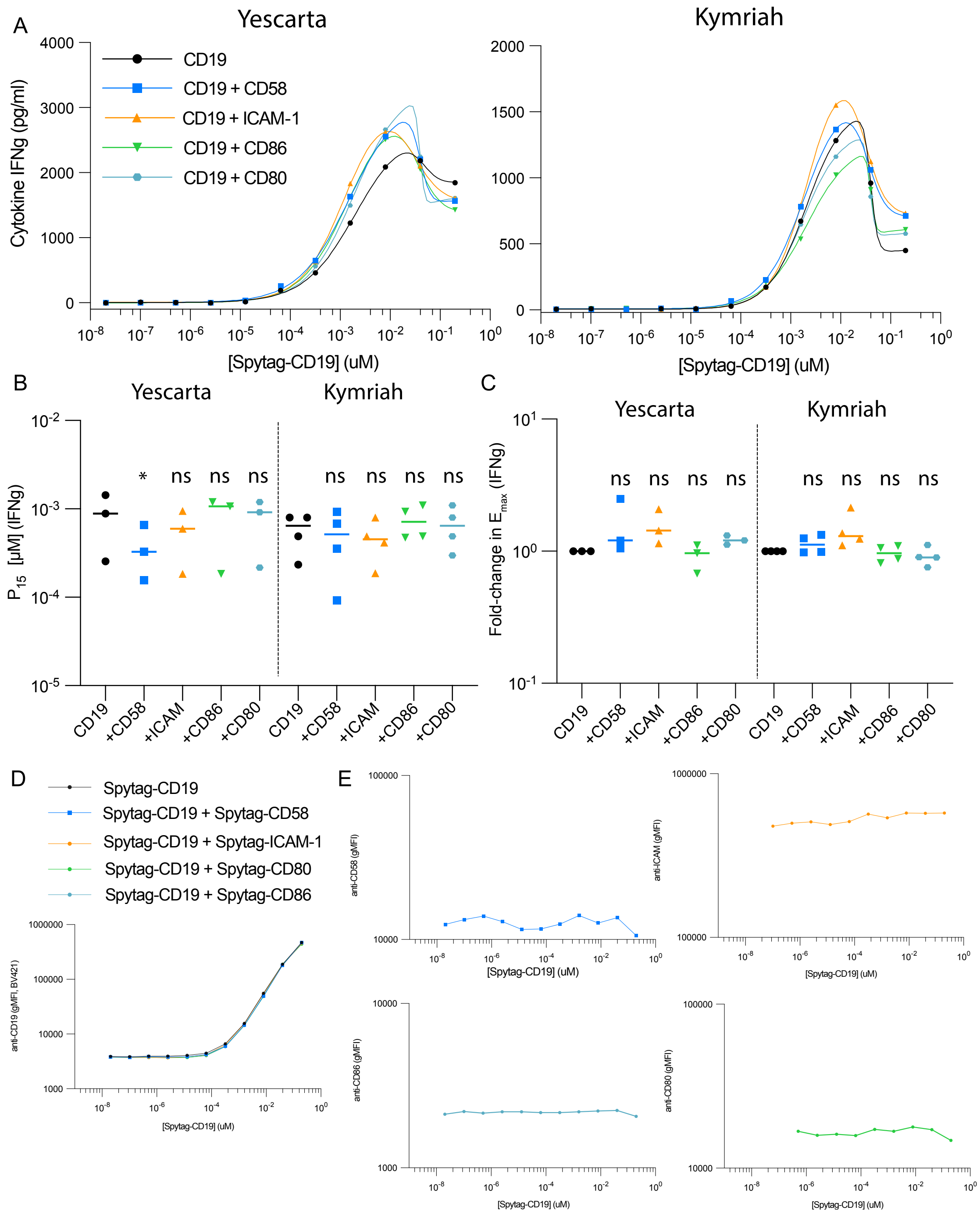

Appendix Figure S3: **Cytokine production by CD19-targeting CAR-T cells is largely independent of accessory receptors (Related to Figure 4E-F).**
(A-C) Cytokine production (A-C) and ligand loading data (D-E) for the N=7 independent experiments (3 for Yescarta and 4 for Kymriah) described in Figure 4E-F. **(A)** Representative dose-response showing a model fit (solid line) used to estimate **(B)** the concentration of Spytag-CD19 required to elicit 15% of the maximal cytokine level (P_{15}) and **(C)** the maximal cytokine level (shown as a fold-change relative to Spytag-CD19 alone). **(D)** Surface levels of Spytag-CD19 and **(E)** each Spytag-ligand on CHO-K1 CombiCells showing that Spytag-ligands do not impact Spytag-CD19 and vice versa. Representative surface levels out of N=7 independent experiments. The concentration of each Spytag-ligand is 0.1 μM and the concentration of Spytag-CD19 as indicated on the x-axes.
Data information: In (B,C), a one-way ANOVA with Sidak's multiple comparison correction was used to determine p-values. Abbreviations: * = p-value$\leq$0.05.

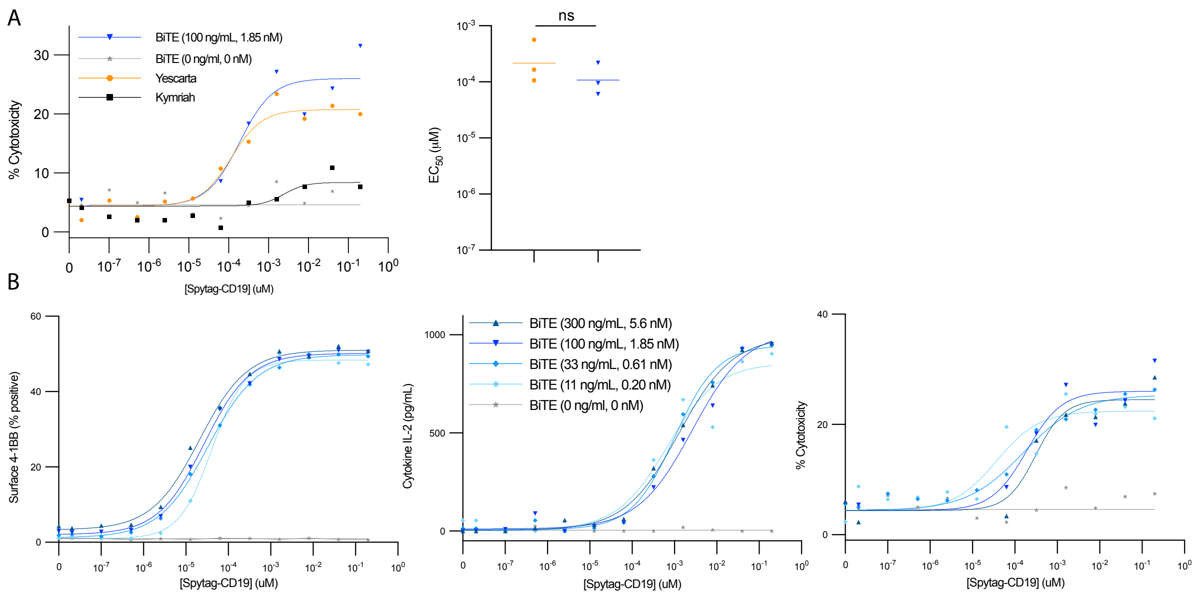

Appendix Figure S4: **The antigen sensitivity of Blinatumomab (BiTE) and Yescarta (CAR) are similar for cytotoxicity.**

(A) Co-cultured of untransduced CD8$^+$ T cells and CD19 KO Nalm6 CombiCells loaded with different concentrations of Spytag-CD19 with the indicated concentration of BiTE. Kymriah and Yescarta transduced CD8$^+$ T cells were also included for comparison. A representative experiment (left) and summary EC_{50} values (right) for N=2 independent experiments. The antigen sensitivity for Kymriah could not be determined because cytotoxicity was similar to background (0 nM of BiTE).

(B) Surface 4-1BB (left), IL-2 secretion (middle), and Cytotoxicity (right) for different concentrations of the BiTE in response to a titration of Spytag-CD19 on the CD19 KO Nalm6 CombiCells.

Data information: In (A), a paired t-test on log-transformed values is used to determine statistical significance.

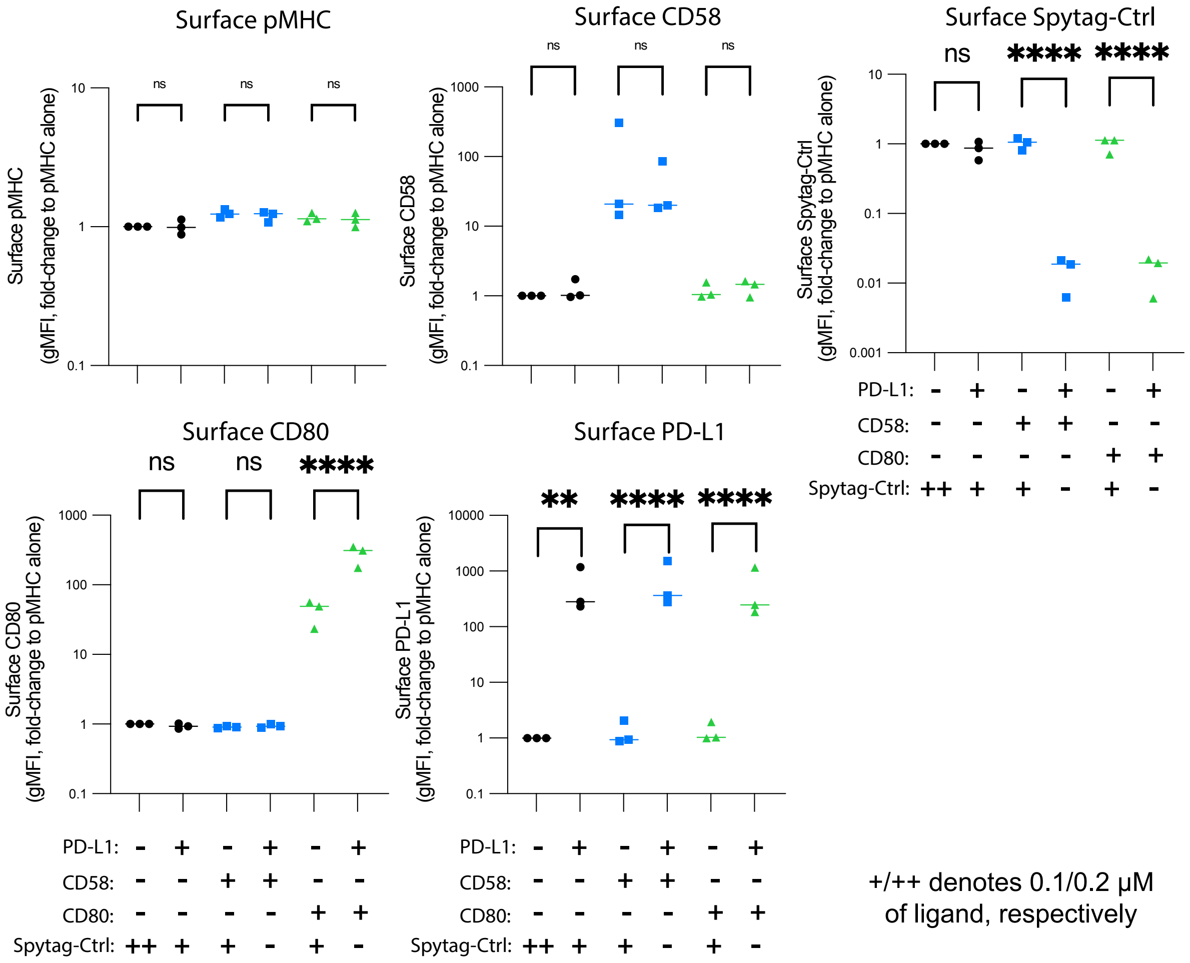

Appendix Figure S5: **Coupling of PD-L1 does not reduce the coupling of pMHC or other ligands, and vice versa, on CHO-K1 CombiCells.**

CHO-K1 CombiCells were coupled with 0.1 μM of the indicated ligands and surface expression of each ligand was detected using antibodies in flow cytometry. The concentration of pMHC in all panels is 0.1 μM and data is presented as fold-changes relative to the gMFI of the pMHC alone condition (first column without PD-L1, CD58, or CD80). The protein CD19 fused to Spytag (Spytag-Ctrl) was added so that the total concentration of additional ligand was always 0.2 μM. Data is shown for N=3 independent experiments.

Data information: A one-way ANOVA with Sidak's multiple comparison correction was used to determine p-values. Abbreviations: * = p-value$\leq$0.05, ** = p-value$\leq$0.01, *** = p-value$\leq$0.001, **** = p-value$\leq$0.0001
