# Supplementary material for: Using CombiCells, a platform for titration and combinatorial display of cell surface ligands, to study T-cell antigen sensitivity modulation by accessory receptors
Source: EMBO J. 2023 Dec 18;43(1):7. doi: 10.1038/s44318-023-00012-1 (PMC10897201; doi:10.1038/s44318-023-00012-1)
Supplement: Supplementary file 9 — Expanded View Figures [file 44318_2023_12_MOESM9_ESM.pdf]

Expanded View Figures

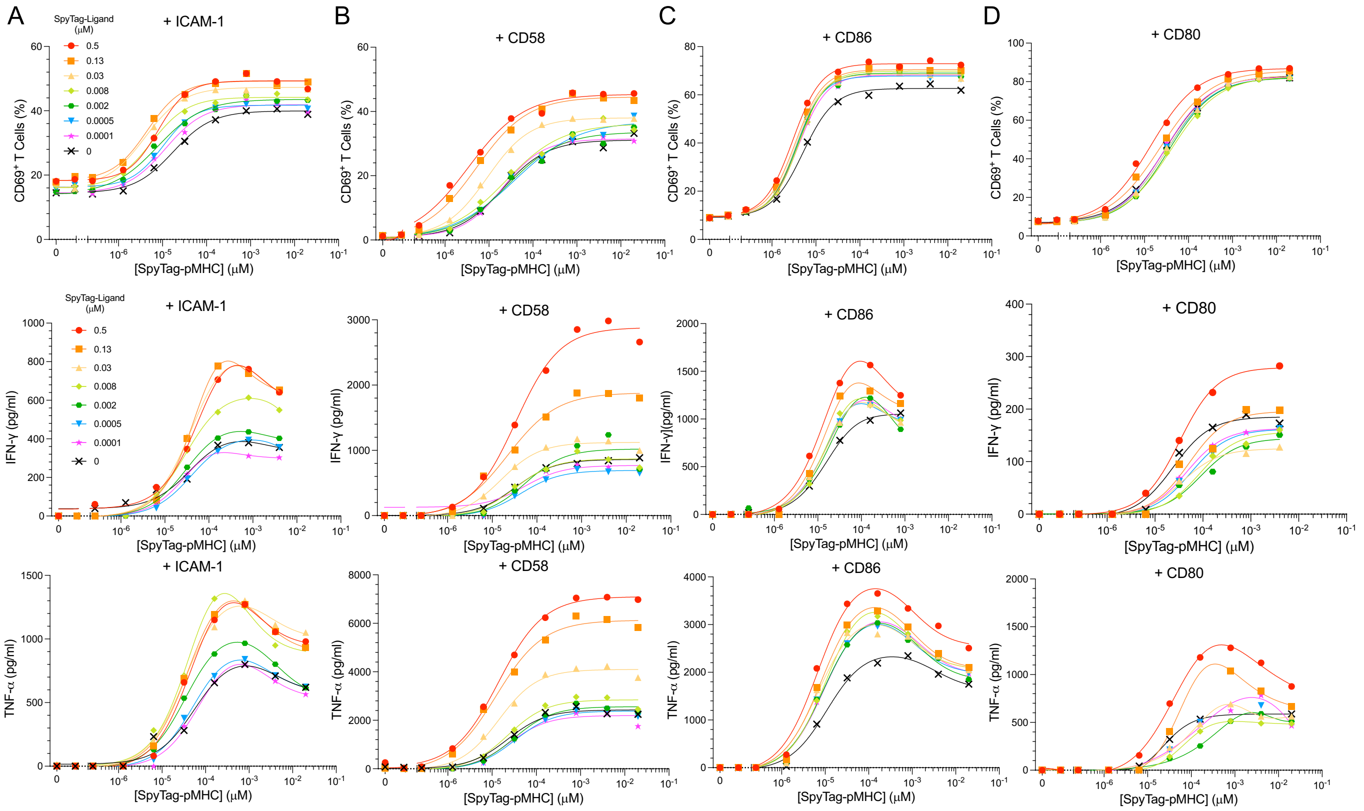

Figure EV1. Additional measures of T cell activation.

Additional measures of T cell activation when coupling (**A**) ICAM-1, (**B**) CD58, **C**) CD86, or (**D**) CD80 on CHO-K1 CombiCells along with pMHC (related to Fig. 2).

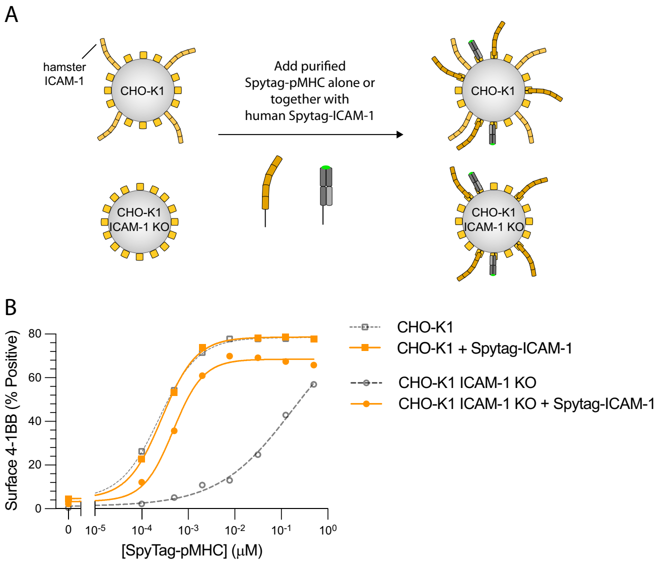

Figure EV2. T cells can exploit endogenously expressed hamster ICAM-1 or exogenous human SpytagICAM-1 when recognizing Spytag-pMHC.

(**A**) Schematic of CHO-K1 cell lines used. (**B**) T cell activation measured by the surface marker 4-1BB in response to Spytag-pMHC alone or in combination with 0.5 μM of Spytag-ICAM-1 on the indicated CHO-K1 cell line.

 © The Author(s)

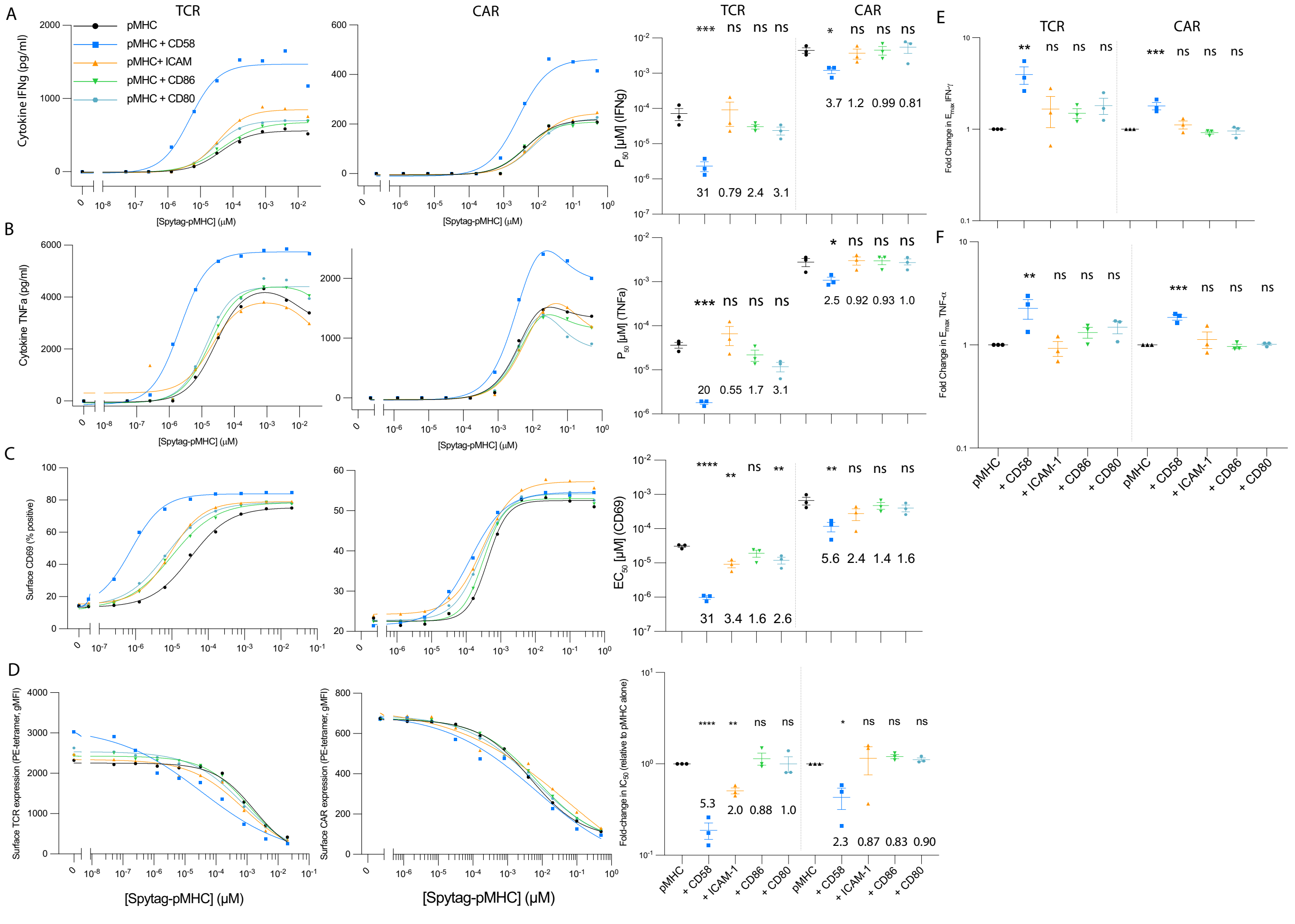

Figure EV3. Additional measures of T cell activation (related to Fig. 3).

(**A–D**) Representative dose-response for the TCR and CAR (left) and summary measures of antigen sensitivity for $N = 3$ independent experiments (right) for (**A**) IFN-γ, (**B**) TNF-α, (**C**) surface CD69, and (**D**) surface antigen receptor. (**E**, **F**) The fold-change in the maximum (**E**) IFN-γ and (**F**) TNF-α relative to pMHC antigen alone from $N = 3$ independent experiments. Data information: In (**A–D**, right panels) and (**E**) the mean and SEM are shown. A *t* test with Dunnett's multiple comparison correction on log-transformed values is used to determine *p* values. **p* value ≤ 0.05, ***p* value ≤ 0.01, ****p* value ≤ 0.001, *****p* value ≤ 0.0001.

© The Author(s)

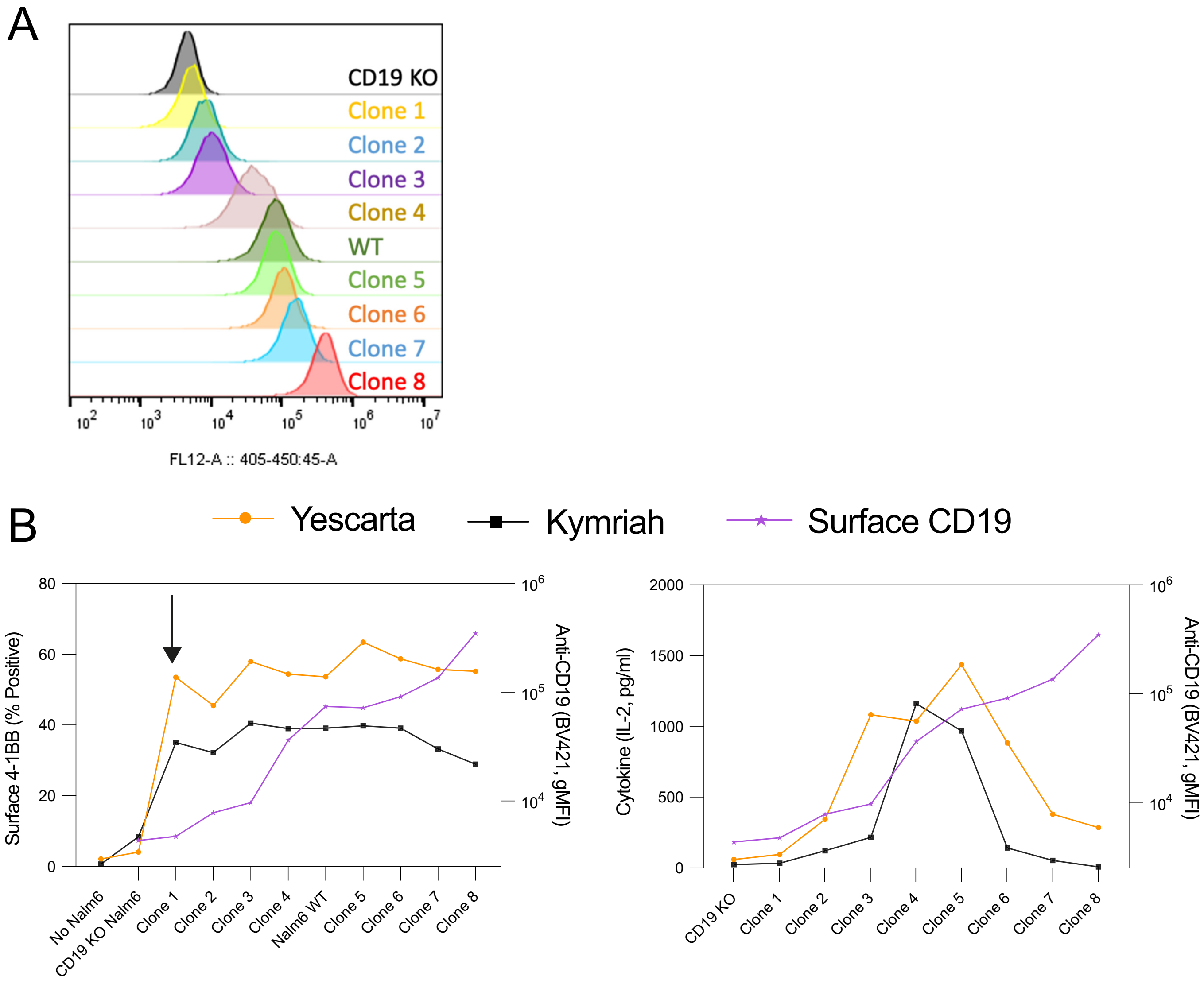

Figure EV4. CAR-T cells recognizing CD19 endogenously expressed at different levels on the surface of a panel of Nalm6 cell lines.

(**A**) Surface expression of CD19 on the indicated Nalm6 clone. (**B**) Primary human CD8$^+$ T cells were co-cultured with the indicated Nalm6 clone for 6 h before T cell activation was assessed by surface 4-1BB (left) and the supernatant levels of IL-2 (right). The complete activation of 4-1BB is observed in response to the Nalm6 cell line expressing the lowest level of CD19 (Clone 1, see right *y*-axes for CD19 level on each Nalm6 cell line). Data information: A representative experiment out of 2 independent experiments is shown.

 © The Author(s)

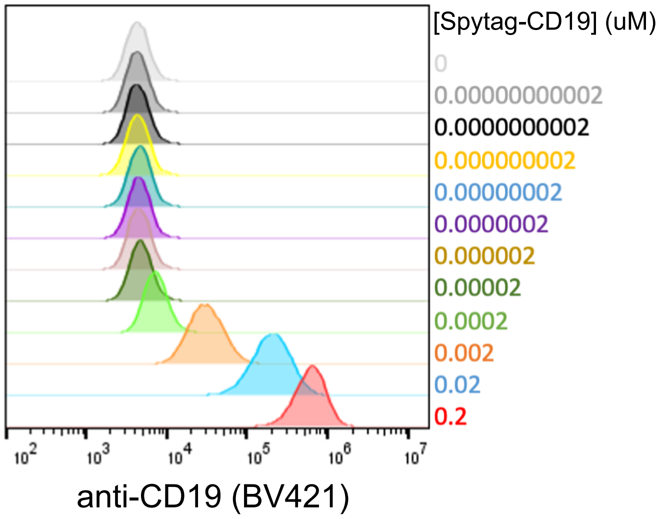

Figure EV5. Variation in surface levels of CD19 on Nalm6 CombiCells produced by titration of Spytag-CD19.

The indicated concentration of purified Spytag-CD19 was coupled to Nalm6 CombiCells before being detected by flow cytometry. Data information: A representative example out of 3 independent experiments is shown.
